# Masking reveals parallel form systems in the visual brain

**DOI:** 10.3389/fnhum.2014.00567

**Published:** 2014-07-25

**Authors:** Yu Tung Lo, Semir Zeki

**Affiliations:** Wellcome Laboratory of Neurobiology, Department of Cell and Developmental Biology, University College LondonLondon, UK

**Keywords:** visual masking, form perception, hierarchical model, parallel processing

## Abstract

It is generally supposed that there is a single, hierarchically organized pathway dedicated to form processing, in which complex forms are elaborated from simpler ones, beginning with the orientation-selective cells of V1. In this psychophysical study, we undertook to test another hypothesis, namely that the brain’s visual form system consists of multiple parallel systems and that complex forms are other than the sum of their parts. Inspired by imaging experiments which show that forms of increasing perceptual complexity (lines, angles, and rhombuses) constituted from the same elements (lines) activate the same visual areas (V1, V2, and V3) with the same intensity and latency (Shigihara and Zeki, 2013, 2014), we used backward masking to test the supposition that these forms are processed in parallel. We presented subjects with lines, angles, and rhombuses as different target-mask pairs. Evidence in favor of our supposition would be if masking is the most effective when target and mask are processed by the same system and least effective when they are processed in different systems. Our results showed that rhombuses were strongly masked by rhombuses but only weakly masked by lines or angles, but angles and lines were well masked by each other. The relative resistance of rhombuses to masking by low-level forms like lines and angles suggests that complex forms like rhombuses may be processed in a separate parallel system, whereas lines and angles are processed in the same one.

## INTRODUCTION

It is widely believed that the form pathway of the visual brain consists of a single hierarchical system beginning in the primary visual cortex (V1) and extending from it to intermediate areas such as V3 and V4 and then on to higher visual areas for further processing (Felleman and Van Essen, 1991; Riesenhuber and Poggio, 1999). Crucial to this pathway are the orientation-selective (OS) cells, which constitute such a conspicuous population of cells in V1, as well as in V2, and the V3 complex (Hubel and Wiesel, 1962, 1965, 1968; Zeki, 1978; Zeki and Shipp, 1988). Even in spite of the abundant reciprocal connections between these areas (Zeki and Shipp, 1988; Felleman and Van Essen, 1991), as well as the documented direct input to them from the lateral geniculate nucleus (Fries, 1981; Yukie and Iwai, 1981) and the pulvinar (Cragg, 1969; Benevento and Standage, 1981; Leh et al., 2008; Baldwin et al., 2012), this pathway is usually characterized as a hierarchical pathway beginning in V1, implying that one stage of the pathway is critically dependent on the antecedent stage and in turn determines the response of the succeeding one.

Recent imaging experiments (Shigihara and Zeki, 2013, 2014) and previous clinical evidence have left us wondering whether this hierarchical strategy is supplemented by a parallel strategy. The imaging experiments, based on recording the latency and strength of activity in visual areas V1, V2, and V3 when subjects viewed stimuli of increasing complexity (lines, angles, and rhomboids) but constituted from the same straight (oriented) lines, show that the three areas are activated with similar strengths and latencies, with angles producing the strongest and rhomboids the weakest responses in all three. Antecedent clinical evidence also shows that agnosia for lines is not necessarily accompanied by agnosia for more complex forms (Hiraoka et al., 2009), thus hinting at a parallel processing of forms of different complexity.

To pursue these findings, we thought it interesting to conduct psychophysical experiments using backward masking to determine whether the evident perceptual hierarchy in the progression from lines to more complex forms such as angles and rhombuses constituted from them is mirrored by a differential susceptibility to masking between forms of differing complexity. Backward masking is a psychophysical technique that involves showing two stimuli, separated by a brief time gap, in quick succession. The first pattern is the “target” and the second the “mask.” The task is to identify the target following the presentation of the mask. Multiple studies (Macknik and Livingstone, 1998; Breitmeyer and Öǧmen, 2006; Breitmeyer, 2008) have shown that when the target is presented alone, or is followed by the mask after a long delay (e.g., 500 ms), it can be easily identified. However, when the mask is presented after a brief delay (e.g., 30 ms) following the target, the target can be rendered imperceptible. Backward masking is therefore capable of interrupting on-going processing activities, preventing them from becoming perceptually explicit.

Masking is most effective if the mask evokes activity in the same processing system as the target (Turvey, 1973; Enns, 2004) or if the mask is presented to the same eye after the target rather than dichoptically, since the latter engages separate visual pathways. Pattern masks, however, remain effective even when presented dichoptically, confirming the view that masking by more complex stimuli happens at the cortical level, where most cells (outside of V1) are binocular (Zeki, 1978). Furthermore, Cheadle and Zeki (2011) demonstrated differential masking between different cortical systems, namely the color and motion systems, which are the two systems that are the most obviously separate in their neural pathways and processing times (Zeki, 1978; Livingstone and Hubel, 1987; Moutoussis and Zeki, 1997; Viviani and Aymoz, 2001; Clifford et al., 2003; Arnold, 2005, *inter alia*) as well as between faces and non-faces (Loﬄer et al., 2005).

These studies encouraged us to use the backward masking paradigm to enquire whether differential masking can also be observed within a single system concerned with a specific visual attribute, namely form. We tested the hypothesis that geometric forms constituted from the same line segments belong in separate categories and are processed by different systems or by pathways operating in parallel to one another.

## MATERIALS AND METHODS

We investigated the differential effects of masking between lines, angles, and rhombuses. Due to the length of the experiments, which lasted on average 1.5 h, we carried out three separate experiments on different subjects: Experiment 1 compared the effectiveness of masking between angles and rhombuses, Experiment 2 that between lines and angles, and Experiment 3 that between lines and rhombuses.

### SUBJECTS

A total of 26 subjects (12 males and 14 females) participated in the 3 Experiments. There were eight subjects aged 18–31 years (three males and five females) in Experiment 1, eight subjects aged 23–26 years (six males and two females) in Experiment 2, and 10 subjects aged 19–28 years (three males and seven females) in Experiment 3. Different subjects were recruited for each experiment, except for one of the authors (YTL) who participated in all three. All subjects had normal or corrected-to-normal vision (visual acuity was not formally assessed, although subjects had to fill in a screening questionnaire affirming they have normal or corrected-to-normal vision prior to taking part in the experiment). The study was approved by the Ethics Committee of University College London. All subjects consented to participating in the experiments and were paid at the end of the experiment.

### APPARATUS

A 16-inch (406 mm) Sony Trinitron Multi-Scan G520 CRT screen was used for the three experiments. The screen has display dimensions of 1024 × 768 pixels and a refresh rate of 60 Hz; it was placed 480 mm from the subjects, who viewed it with their heads on an adjustable chin-rest and their eyes roughly level with the center of the screen. The three experiments were performed in a standard psychophysics dark room.

A photometer (PhotoResearch Spectra-Colorimeter Model PR-670) was used to measure the luminance of the stimuli and the background. Cogent 2000 Toolbox for MATLAB (http://www.vislab.ucl.ac.uk/cogent_2000.php) was used to execute the scripts for these experiments.

### STIMULI

The target and mask stimuli each consisted of 60 figures; each figure could be (a) four parallel lines of length 1° and thickness of 0.05°; (b) a pair of opposing angles constituted from lines of the same length and thickness as (a); or (c) a rhombus, constituted from the same lines as in (a). The lines constituting all stimuli were white (luminance of 333 cd/m^2^), against a gray background (luminance of 65 cd/m^2^).

**FIGURE 1:**
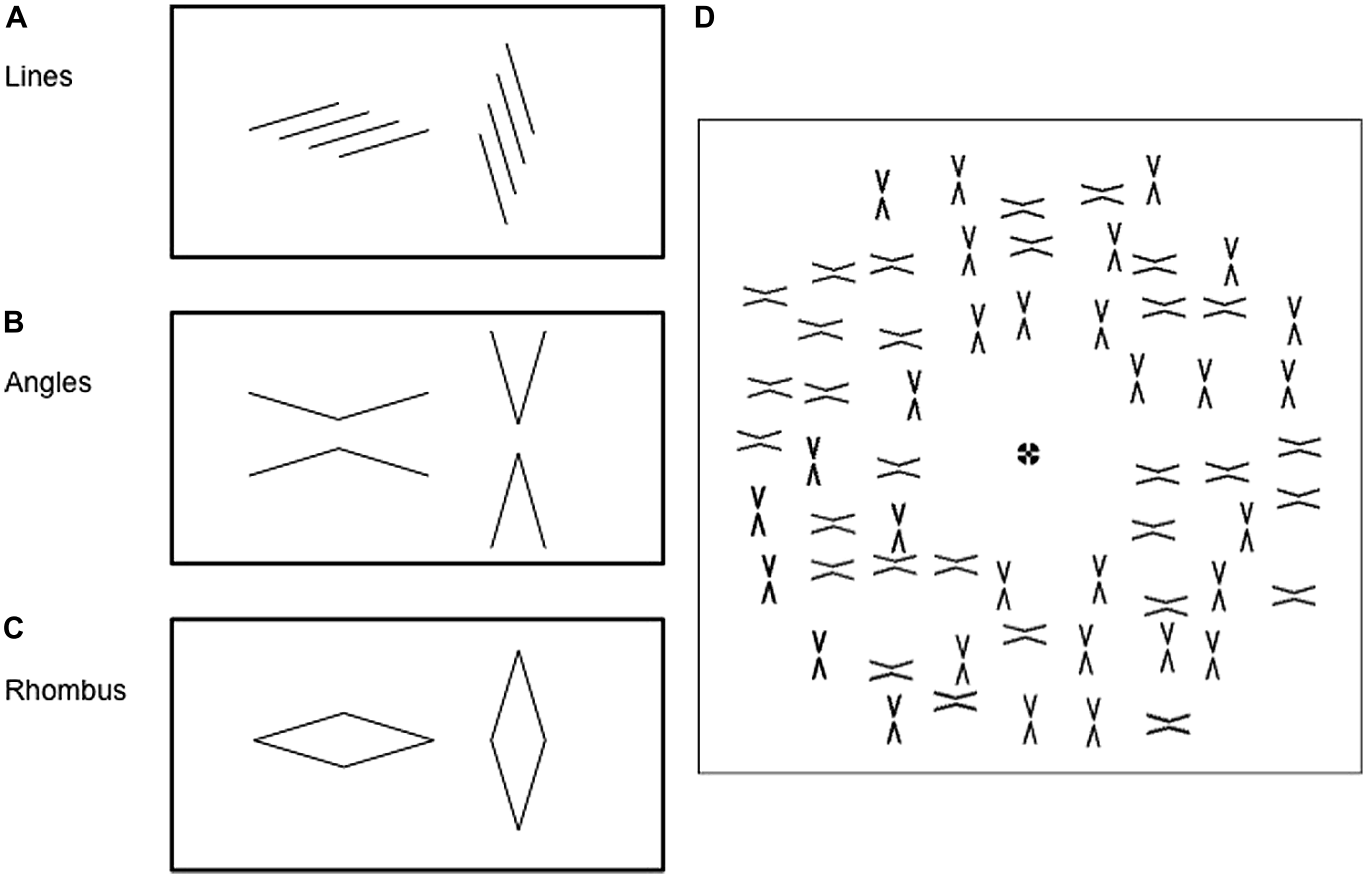
**Each of the three types of figures may be presented in 2 configurations: (A) four parallel lines arranged with either a leftward slant or a rightward slant: **(B)** a pair of obtuse (150°) or acute (30°) angles; or **(C)** rhombuses in either a horizontal and vertical configuration. (D)** Figures were arranged in an annular pattern around a central fixation point. The angle mask pattern is used as an example here. Only one configuration was shown as the target pattern (not shown), whereas a mixture of the two configurations was shown as the mask pattern, as in this example.

Each figure could take on two geometric configurations (**Figure 1**). Line stimuli consisted of four parallel lines with either a leftward or rightward slant (**Figure 1A**). Angle stimuli consisted of two opposing obtuse (150°) or acute (30°) angles, with a 0.3° gap between their vertices (**Figure 1B**). Finally, rhombus stimuli were oriented either horizontally or vertically (**Figure 1C**). In each category of stimulus, the figures were arranged in a non-overlapping manner in an annulus of inner radius of 3° and outer radius of 16° (**Figure 1D**); the figures were spaced between 2.5 and 3.5° from one another. A fixation point was shown in the center of the annulus. Subjects were instructed to look at the fixation point throughout the experiment.

Similar configurations were used for both target and mask stimuli except that, in the mask stimulus, half of the figures were orientated in one configuration and half in the other (**Figure 1D**), while in the target stimulus all figures had the same configuration. The use of an array of figures and a mixture of configurations in the mask pattern was to de-emphasize the role of structural similarities between target and mask. For instance, a left-slanting line could, in principle, mask another left-slanting line more effectively than a right-slanting line, purely due to a greater degree of spatial overlap. Having multiple figures in the mask pattern allowed some left-slanting lines to be masked by left-slanting lines or by right-slanting ones.

**FIGURE 2:**
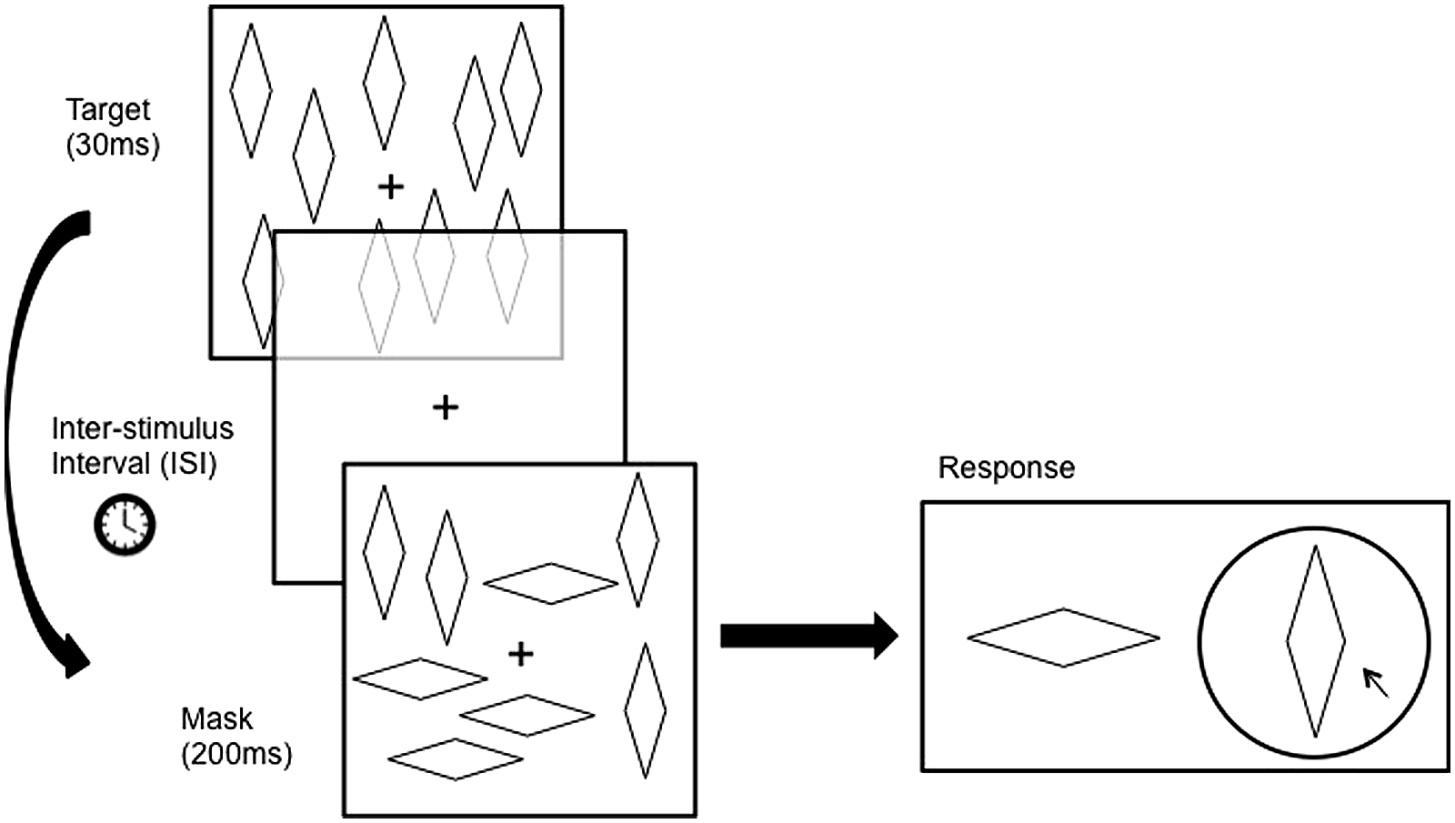
**Atypical trial Involves the presentation of a target pattern for a duration of 30 ms followed by a blank screen for a duration (Inter-stimulus Interval, ISl) of either 17 ms, 100 ms, or (In Experiment 1 only) 500 ms, followed by the presentation of the mask pattern for 200 ms.** The target pattern contains figures in only one configuration, whereas the mask pattern contains a mixture of both configurations. Subjects then had to report which of the wo configurations was seen in the target pattern. Figures are not drawn to scale here.

**Figure 2** illustrates the sequence of a typical trial. The mask was presented after the target (backward masking) with three possible inter-stimulus intervals (ISI, the interval between the termination of the target and the onset of the mask): 17 ms, 100 ms, and 500 ms. The target and mask were presented for 30 ms and 200 ms, respectively. The ISIs were applied in a random order over each block. There was a 1000 ms delay between the response and the appearance of the next target, and a 500 ms delay between the termination of the mask and the presentation of the response screen. Only the fixation point remained on the screen during these periods. Subjects were then asked to identify which of the two configurations was presented as the target pattern.

Experiment 1 consisted of eight blocks of 120 trials (40 trials for each of the three ISI). Experiment 2 and 3 omitted the 100 ms ISI conditions, and therefore consisted of eight blocks of 80 trials (40 trials for each of the remaining two ISI). The eight blocks were divided into two groups of four blocks. Each group of 4 consisted of the 4 possible target-mask combinations done in a random order. Each target-mask combination was therefore presented twice over the eight blocks. For each of the eight blocks, subjects completed a variable number of practice trials until they were ready to begin. After the first four blocks subjects took a break for 5 min.

### HYPOTHESIS

**FIGURE 3:**
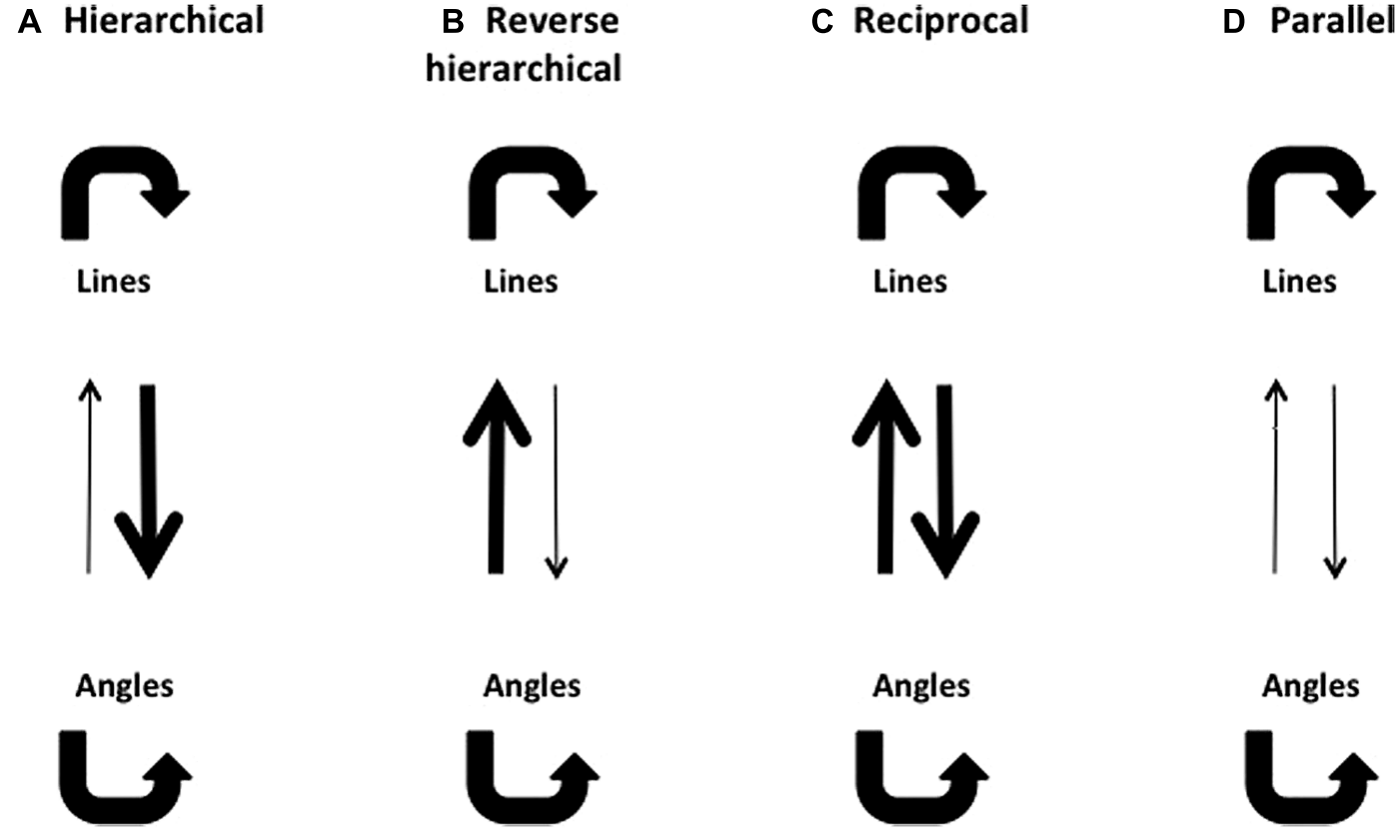
**Possible masking patterns, using lines and angles as examples.** Thick arrows denote strong masking effects, and thin arrows denote weak masking effects. **(A)** A hierarchical model predicts that lines should be able to mask angles, but angles should not be able to mask lines. **(B)** A reverse hierarchical model predicts that angles should be able to mask lines, but lines should not be able to mask angles. **(C)** A reciprocal model predicts that lines and angles should be able to mask each other effectively. **(D)** A parallel model predicts that neither lines nor angles should be able to mask each other effectively.

As shown in **Figure 3A**, the hierarchical model predicts that a complex pattern would be masked by simpler patterns lower in the hierarchy and processed in the same visual pathway. For example, rhombuses should be effectively masked by angles and lines, whereas angles should be well masked by lines and angles but not by rhombuses, By the same token, lines should only be well masked by lines but not angles or rhombuses. This would be so if each system were completely isolated, with no cross talk between the systems. In reality there are many recurrent and feedback connections which could influence responses. For example, in **Figure 3B**, we show a possible schema in which a complex figure masks a simpler one through feedback connections (indeed we did obtain a result that can be interpreted in this way). Equally, two forms could conceivably mask each other with equal strength because of reciprocal connections between them (which could interpret another one of our results), as shown in **Figure 3C**. On the other hand, the parallel processing model would predict that masking is the strongest when target and mask are both processed in the same visual pathway, and weakest when they are processed in separate pathways. For example, only rhombuses should mask rhombuses and not lines or angles while angles should be well masked only by angles but not by lines or rhombuses, and lines should be well masked only by lines but not by angles or rhombuses. A hypothetical masking pattern for the parallel processing model is shown in **Figure 3D**.

## RESULTS

Some target and mask combinations were much more effective in masking the perception of the target than others. In general, the most effective mask was one that belonged to the same category as the target stimulus. We also observed a relative sparing of rhombus perception by line or angle masks.

### EXPERIMENT 1: ANGLES AND RHOMBUSES

**FIGURE 4:**
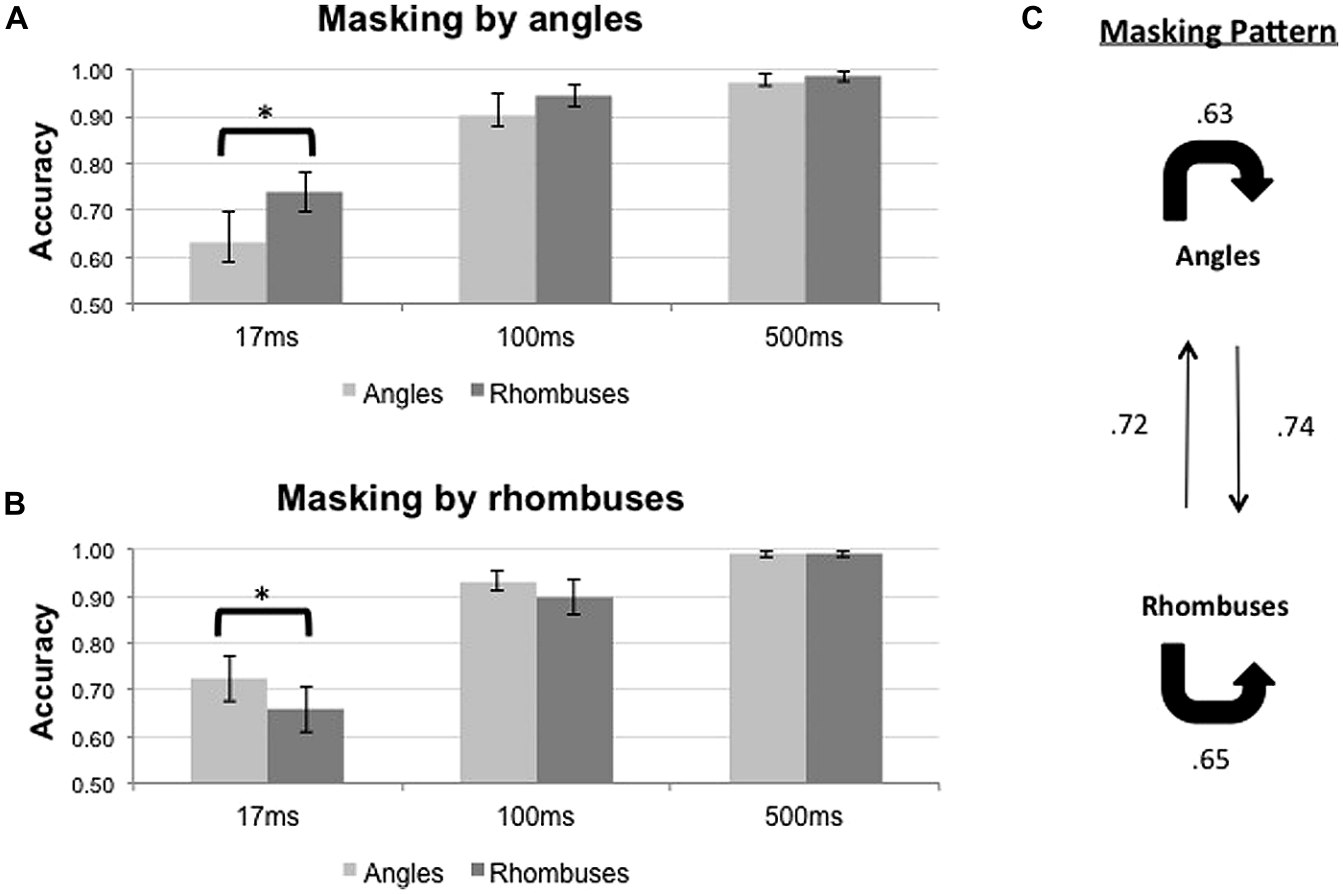
**Proportion of correct responses (*n* = 8) when angles (light gray bars) or rhombuses (dark gray bars) were masked by **(A)** angles or **(B)** rhombuses, across three ISI settings (17, 100, and 500 ms).** Differential masking effect was demonstrated between angles and rhombuses In the 17 ms condition. Chance level is at 0.50. Error bars denote standard errors of means. Asterisks denote statistically significant difference between conditions as shown by paired samples *t-*test. **(C)** Masking pattern between angles and rhombuses. The accuracy of target identification was indicated by the numbers next to the arrows.

The results of the backward masking for angles and rhombuses are shown in **Figure 4**. **Figure 4A** shows the results with angle masks while **Figure 4B** shows the results with rhombus masks. The overall masking pattern between angles and rhombuses is summarized in graphical form in **Figure 4C**.

For each mask type, Repeated Measures ANOVAs were used to determine the effects of ISI and target type on the accuracy of target identification. Differential masking effects exist when a given mask type consistently suppresses the identification of one target type more than the other. The results are summarized in **Table 1**. The duration of the ISI significantly affected the accuracy for both mask types [*F*_2,14_ = 25.547, *p* < 0.001 for angle masks, and *F*_2,14_ = 26.685, *p* < 0.001 for rhombus masks]. Pooling together all three ISI conditions, we did not observe any differential masking effect [*F*_1,7_ = 4.821, *p* = 0.064 for angle masks and *F*_1,7_ = 8.775, *p* = 0.021 for rhombus masks]. Differential masking effects were, however, observed after also accounting for the ISI [Target × ISI interaction, *F*_2,14_ = 4.435, *p* = 0.032 for angle masks, *F*_2,14_ = 4.006, *p* = 0.042 for rhombus masks]. Only the 17 ms ISI conditions showed a differential masking effect – angles masked angles more effectively than rhombuses [mean difference in accuracy = -0.106, *t*(7) = -2.51, *p* = 0.041], and conversely rhombuses masked rhombuses more effectively than angles [mean difference in accuracy = -0.066, *t*(7) = -2.74, *p* = 0.029]. In addition, performances were nearly perfect for the 500 ms ISI conditions (accuracy >0.98 for all), hence indicating the absence of confounding factors such as different visibility between target types (or mask types), inattention, general task difficulty, and confusion about instructions. We therefore omitted the 100 ms conditions in our subsequent experiments, as the 500 ms ISI conditions were sufficient to serve as positive controls.

**Table 1:** Repeated Measures ANOVA and *post hoc* pairwise comparisons for Experiment 1 (*n = 8*).

	ANOVA	*P*	*Partial*η^2^	*Post hoc* pairwise comparison, *P*
ANOVA (masking by angles) Within-subject effects				
ISI	*F*_2,14_ = 25.547	<0.001	0.785	
17 vs. 100 ms				0.009*
17 vs. 500 ms				0.002*
100 vs. 500 ms				0.124
	*F*_1,7_ = 4.821	0.064	0.408	
Target × ISI	*F*_2,14_ = 4.435	0.032	0.388	
Angle vs. rhombus (17 ms ISI)				0.041*
Angle vs. rhombus (100 ms ISI)				0.220
Angle vs. rhombus (500 ms ISI)				0.359
ANOVA (masking by rhombuses) Within-subject effects				
ISI	*F*_2,14_ = 26.685	<0.001	0.792	
17 vs. 100 ms				0.008*
17 vs. 500 ms				0.001*
100 vs. 500 ms				0.085
Target	*F*_1,7_ = 8.775	0.021	0.556	
Target × ISI	*F*_2,14_ = 4.006	0.042	0.364	
Angle vs. rhombus (17 ms ISI)				0.029*
Angle vs. rhombus (100 ms ISI)				0.080
Angle vs. rhombus (500 ms ISI)				1.000

*Bonferroni correction is applied for all multiple comparisons. ^∗^Statistically significant*p*-values are marked with an asterisk.*

In summary, a symmetrical differential masking effect was observed between angles and rhombuses (**Figure 4C**), suggesting that they are processed by parallel systems.

### EXPERIMENT 2: LINES AND ANGLES

**FIGURE 5:**
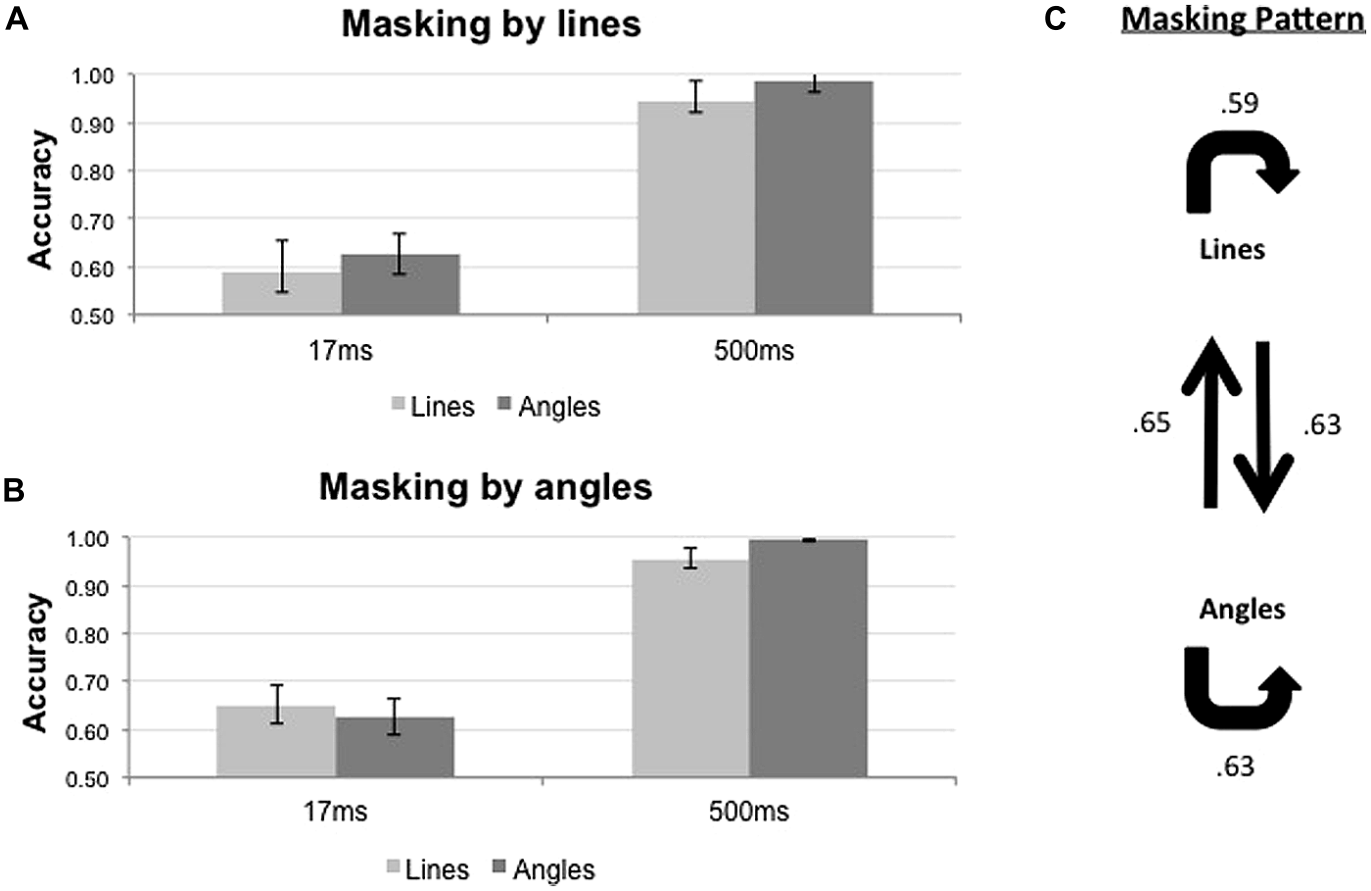
**Proportion of correct responses (*n* = 8) when lines (light gray bars) or angles (dark gray bars) were masked by **(A)** lines or **(B)** angles, across two ISI settings (17 and 500 ms). (C)** Masking pattern between lines and angles. Conventions as in ****Figure 4****.

The results of the backward masking for lines and angles are shown in **Figure 5**. **Figure 5A** shows the results with line masks, and **Figure 5B** shows the results with angles masks. The overall masking pattern between lines and angles is summarized in graphical form in **Figure 5C**.

The results of statistical analyses are summarized in **Table 2**. For both mask types, only ISI influenced the accuracy of target identification [*F*_1,7_ = 61.843, *p* < 0.001 for line masks, and *F*_1,7_ = 85.897, *p* < 0.001 for angle masks]. We did not observe any significant differential masking effect for both line masks [Target × ISI; *F*_1,7_ = 0.042, *p* = 0.844] and angle masks [Target × ISI; *F*_1,7_ = 2.692, *p* = 0.145] in the 17 ms ISI condition

**Table 2:** Repeated Measures ANOVA and post hoc pairwise comparisons for Experiment 2 (n = 8).

	ANOVA	*P*	*Partial*η^2^	*Post hoc* pairwise comparison, *P*
ANOVA (masking by lines)Within-subject effects				
ISI	*F*_1,7_ = 61.843	<0.001	0.898	
17 vs. 500 ms				<0.001*
Target	*F*_ 1,7_ = 2.983	0.128	0.299	
Target × ISI	*F*_1,7_ = 0.042	0.844	0.006	
ANOVA (masking by angles)Within-subject effects				
ISI	*F*_1,7_ = 85.897	<0.001	0.925	
17 vs. 500 ms				<0.001*
Target	*F*_1,7_ = 0.075	0.792	011	
Target × ISI	*F*_1,7_ = 2.692	0.145	0.278	

*Bonferroni correction is applied for all multiple comparisons. ^∗^Statistically significant p-values are marked with an asterisk.*

In summary, lines and angles masked each other to the same extent and there was no evidence that lines and angles belong to different systems (**Figure 5C**).

### EXPERIMENT 3: LINES AND RHOMBUSES

**FIGURE 6:**
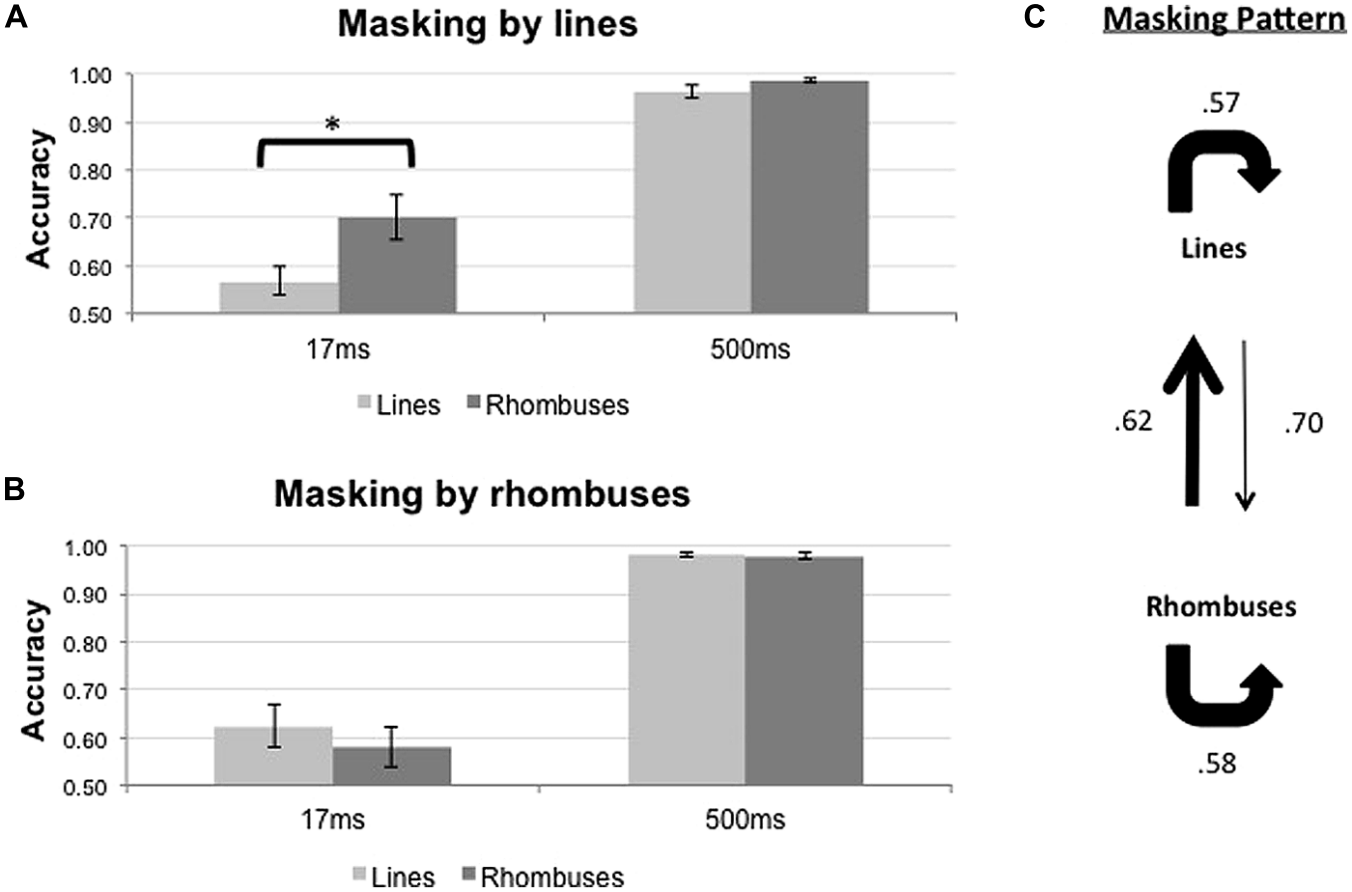
**Proportion of correct responses (*n* = 10) when lines (light gray bars) or rhombuses (dark gray bars) were masked by **(A)** lines or **(B)** rhombuses, across two ISI settings (17 and 500 ms). (C)** Masking pattern between lines and rhombuses. Conventions as in ****Figure 4****.

The results of the backward masking for lines and rhombuses are shown in **Figure 6**. **Figure 6A** shows the results with line masks, and **Figure 6B** shows the results with rhombus masks. The overall masking pattern between lines and rhombuses is summarized in graphical form in **Figure 6C**.

The results of statistical analyses are summarized in **Table 3**. For both mask types, ISI influenced the accuracy of target identification [*F*_1,9_ = 69.922, *p* < 0.001 for line masks, and *F*_1,9_ = 86.679, *p* < 0.001 for rhombus masks]. Differential masking effect occurred when lines were used as masks [Pooled, *F*_1,9_ = 14.790, *p* = 0.004 and Target × ISI interaction, *F*_1,9_ = 18.235, *p* = 0.002], with lines masking lines more effectively than rhombuses [mean difference in accuracy = -0.133, *t*(9) = -4.22, *p* = 0.002]. No differential masking occurred when rhombuses were used as masks [Pooled, *F*_1,9_ = 3.613, *p* = 0.90 and Target × ISI interaction, *F*_1,9_ = 2.045, *p* = 0.186], indicating that rhombuses masked lines as effectively as rhombuses.

**Table 3:** Repeated Measures ANOVA and post hoc pairwise comparisons for Experiment 3 (n = 10).

	ANOVA	*P*	*Partial*η^2^	*Post hoc* pairwise comparison, *P*
ANOVA (masking by lines) Within-subject effects				
ISI	*F*_1,9_ = 69.922	<0.001	0.886	
17 vs. 500 ms				<0.001*
Target	*F*_ 1,9_ = 14.790	0.004	0.622	
Line vs. rhombus (pooled)				0.004*
Target × ISI	*F*_1,9_ = 18.235	0.002	0.670	
Line vs. rhombus (17 ms ISI)				0.002*
Line vs. rhombus (500 ms ISI)				0.108
ANOVA (masking by rhombuses) Within-subject effects				
ISI	*F*_1,9_ = 86.679	<0.001	0.906	
17 vs. 500 ms				<0.001*
Target	*F*_ 1,9_ = 3.613	0.090	0.286	
Target × ISI	*F*_1,9_ = 2.045	0.186	0.185	

*Bonferroni correction is applied for all multiple comparisons. ^∗^Statistically significant *p*-values are marked with an asterisk.*

In summary, there is an asymmetrical relationship between line perception and rhombus perception, with rhombuses strongly masking lines (relative to rhombuses) but lines only weakly masking rhombuses (relative to lines; **Figure 6C**).

**FIGURE 7:**
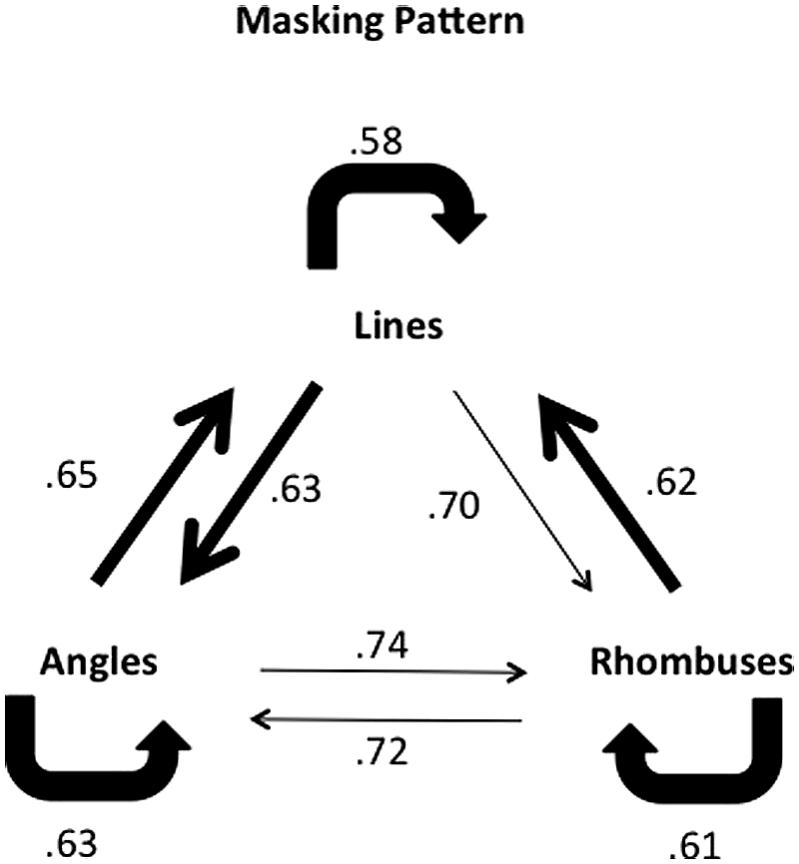
**Combined masking pattern of the three experiments for the 17 ms ISI setting.** The pattern of differential masking for lines and angles suggests that they are processed in the same, possibly recurrent, system. Angles and rhombuses only weakly mask each other. Rhombuses mask lines strongly, but lines mask rhombuses to a lesser extent. Conventions as in previous figures.

The results of all three experiments are summarized in **Figure 7**, and suggest that lines and angles appear to be processed in a common pathway whereas rhombuses appear to be processed in a separate pathway, possibly distinct from that processing angles and lines.

## DISCUSSION

Our results may be summarized as follows:

1. Angles masked angles more effectively than rhombuses and rhombuses masked rhombuses more effectively than angles (Experiment 1).

2. Lines masked angles as effectively as lines, and angles masked lines as effectively as angles (Experiment 2).

3. A differential and asymmetrical masking effect was found to exist between lines and rhombuses since rhombuses masked lines strongly but lines masked rhombuses weakly (Experiment 3).

In the interpretation we give below, we make the assumption that masking constitutes a means of demonstrating parallel processing (Turvey, 1973; Enns, 2004; Loﬄer et al., 2005; Cheadle and Zeki, 2011). We therefore interpret the results of Experiment (1) as indicating that angles and rhombuses are processed in parallel by separate systems; the results of Experiment (2) as indicating that lines and angles are processed by the same system and the results of Experiment (3) as indicating that lines and rhombuses are processed by separate systems. We note that, in this interpretation, when we speak of processing by the same system, we do not necessarily imply that the two forms, for example lines and angles, are hierarchically processed within that system. For example, lines and angles could equally well be processed in parallel within the same system simultaneously, or the processing of lines could precede that of angles, in a hierarchical fashion within that system. However, our separate perceptual experiments (Lo and Zeki, unpublished) indicate that lines and angles are perceived simultaneously, thus strengthening the case for parallel processing.

Overall, our results suggest that lines and angles, which are more or less equally effective in masking each other, constitute one family of forms, while rhombuses, which are more resistant to masking by lines and angles while being able to mask lines, constitute another, separate, form system. This is mirrored by imaging studies (Shigihara and Zeki, 2014) which show that lines and angles activate visual areas V1-V2-V3 with the same strength, and more strongly than rhombuses. Moreover, while V2 angle-selective neurons respond maximally to specific angles, many of them also respond with equal strength to the angles’ linear components (Boynton and Hegdé, 2004; Ito and Komatsu, 2004), indicating that these “angle-selective” neurons do not function as pure angle detectors. In light of this it is not unexpected that lines and angles mask each other to the same extent.

### HIERARCHICAL AND PARALLEL STRATEGIES

When we write of a parallel strategy, we do not mean to imply that it is used exclusively. Rather, we believe that it is used in parallel with a hierarchical strategy. As well, we do not think of a parallel strategy as necessarily using separate visual areas. It is conceivable that the same visual areas may be involved in processing separate forms in parallel. Equally, while we speak broadly of parallel pathways as separate, independent pathways which could operate within the same area(s) or engage different ones, we do not think of them as isolated. More likely, the known rich connections within visual areas and between them, including feedforward and feedback interactions, could play decisive roles in determining the responses of the separate processing systems, each one of which may as well be hierarchically organized. It is possible to account for the masking of lines by the other forms used in this study, for example, by supposing that there is a feedback, which modulates the processing of lines. Such extensive feedback influences from higher level cortical areas to lower ones are known to exist (Rockland and Pandya, 1979; Felleman and Van Essen, 1991; Lamme and Roelfsema, 2000; Murray et al., 2002) and might influence the activity of orientation-selective (OS) cells, known to constitute a significant population of cells in many visual cortical areas (Zeki, 1978, 1983), the OS cells in each area contributing to the processing of forms in different ways. There is extensive anatomical divergence and reconvergence between different visual systems, and very rarely are brain areas completely functionally segregated from one another (DeYoe and Van Essen, 1988; Zeki and Shipp, 1988). Hence we do not assume that each of the parallel system is isolated from other systems and operates with complete independence.

### OS CELLS AND FORMS

The ubiquitous presence of OS cells in areas V1, V2, V3, V3A, and V3B is a puzzle. One answer to why OS cells should be so widely distributed in cortex might be that they are put to different uses, for elaborating different forms, in parallel. It would be hard to conceive of a brain that uses vastly different mechanisms for elaborating the infinite number of forms that we are capable of perceiving. It is far easier to suppose that the same basic mechanism, the OS cell, is put to different use in different areas for elaborating different forms. If so, then one could also hypothesize that the OS cells of V1 are not the only source for constructing forms. Rather, basically the same OS cell is used differently in each of the areas in which it is distributed to construct forms. We do not intend to give this hypothesis too dogmatic a character. But it is worth considering as a possibility in light of the evidence presented here and in imaging studies exploring the activation of visual areas by forms of increasing complexity (Shigihara and Zeki, 2013, 2014), as well as the antecedent evidence showing that the visual areas outside of V1 receive direct inputs from the LGN (Fries, 1981; Yukie and Iwai, 1981) and pulvinar (Cragg, 1969; Benevento and Standage, 1981; Leh et al., 2008; Baldwin et al., 2012), and that they contain heavy concentration of OS cells.

## Conflict of Interest Statement

The authors declare that the research was conducted in the absence of any commercial or financial relationships that could be construed as a potential conflict of interest.
